# Which Risk Factors Matter to Whom?

**DOI:** 10.1371/journal.pmed.0010029

**Published:** 2004-10-19

There is a much-quoted saying, attributed to the epidemiologist Geoffrey Rose: “A large number of people exposed to a small risk may generate many more cases than a small number exposed to a very high risk.” This is true for many individual risk factors such as salt intake (linked to high blood pressure and cardiovascular disease) and speeding on the highway (linked to injuries and accidents). Does it apply to many other global health risks? The study by Anthony Rodgers and colleagues suggests that it does.

To develop effective health policies, one must understand the existing health risks and disease burdens. On a worldwide scale, this is a tough challenge. The Global Burden of Disease Database, maintained by the World Health Organization (WHO), collects data from countries around the world on risk factors such as tobacco, malnutrition, childhood abuse, unsafe sex, childbirth, and cholesterol levels, as well as on disease burdens, for example depression, blindness, and diarrhea. A large group of scientists from all over the world has developed a framework to analyze these data. To compare different risks or burdens, they calculate disability-adjusted life-years, or DALYs—the number of healthy life years lost because of a particular disease or risk factor.[Fig pmed-0010029-g001]

**Figure pmed-0010029-g001:**
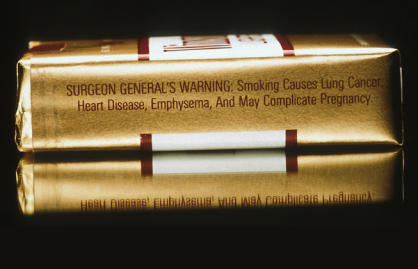
Tobacco is a major player in the global burden of disease (Photo: Bill Branson)

Rodgers and colleagues used data from the WHO database for 26 risk factors and from 14 epidemiological subregions of the world to calculate the proportion of risk-factor-attributable disease burden in different population subgroups defined by age, sex, and exposure level. For being underweight in childhood, for example—the leading risk factor for global loss of healthy life—they found that only 35% of the disease burden occurred in severely underweight children, the rest occurred in those only moderately underweight. The relative risks for the moderately underweight are much lower, but the number of children in that category is so large that the total attributable burden amounted to almost two-thirds of the total global burden of disease for that risk factor.

The analysis confirms—and extends to a global level—previous research showing that many major health risks are important across the range of exposure levels, not just among individuals exposed to high levels of risk. It also points to risk factors that are particularly prevalent among specific populations and age groups, and for which highly targeted interventions could be effective.

Despite numerous caveats and limitations of studies like this one, such analyses are essential aids in guiding the distribution of limited funds to lower the burden of life years lost to premature death and disability.

